# Navigating the path forward: advancing global health in a changing world – the 31st Canadian Conference on Global Health in 2025

**DOI:** 10.7189/jogh.16.02001

**Published:** 2026-07-03

**Authors:** Margaret J Mutumba-Nakalembe, Shawna O’Hearn, Johanna C Manga, Cameal Soverall, Reza Khorramizadeh, Krista Kruja, Noni E MacDonald, Muriel Mac-Seing, Salim Sohani, Kathryn E Tiedje, Kate Enright, Khoaja Khaled, Kathryn Macrae, Kofi Bobi Barimah, Janet Hatcher Roberts, Colleen M Davison, Saad El-Din Hassan, Michelle Amri

**Affiliations:** 1 Africa Institute & Frugal Biomedical Innovations, Schulich School of Medicine & Dentistry, Western University, London, Ontario, Canada; 2 Faculty of Medicine, Dalhousie University, Halifax, Nova Scotia; 3 Independent consultant, Montreal, Québec, Canada; 4 Eventum, Toronto, Ontario, Canada; 5 Endocrinology and Metabolism Research Institute, Tehran University of Medical Sciences, Iran; 6 Evidence Link, Toronto, Ontario, Canada; 7 Department of Pediatrics, Dalhousie University, IWK Health Centre Halifax, Nova Scotia, Canada; 8 School of Public Health, Centre de recherche en santé publique, Université de Montréal, Montréal, Québec, Canada; 9 Canadian Red Cross, Ottawa, Ontario, Canada; 10 Department of Microbiology and Immunology, Bio21 Institute and The Peter Doherty Institute for Infection and Immunity, The University of Melbourne, Melbourne, Victoria, Australia; 11 Western Centre for Bioethics, Faculty of Health Sciences, University of Western Ontario, London, Canada; 12 Food Banks Canada, Toronto, Ontario, Canada; 13 Island Health, Victoria, British Columbia, Canada; 14 Ghana Communication Technology University, Accra, Ghana; 15 WHO Collaborating Centre for Knowledge Translation, Technology Assessment for Health Equity, Bruyere Health Research Institute, University of Ottawa, Ottawa, Ontario, Canada; 16 Department of Public Health Sciences, Queen's University, Kingston, Ontario, Canada; 17 Canadian Association for Global Health, Ottawa, Ontario, Canada; 18 The W. Maurice Young Centre for Applied Ethics, School of Population and Public Health, University of British Columbia, Vancouver, British Columbia, Canada

The 31st Canadian Conference on Global Health (CCGH) was held from 24 to 28 October 2025 at the Halifax Convention Centre in Halifax, Nova Scotia, also known as *Mi’kma’ki*, the ancestral and unceded territory of the *Mi’kmaq* People. The conference was offered in hybrid format (in-person and online) with bilingual delivery (English and French). The theme of CCGH 2025 was ‘Navigating the path forward: advancing global health in a changing world’, while the four subthemes were: ‘Navigating global health governance, financing, collaboration, and trust’; ‘Youth, gender, and inclusive leadership; global health security’: ‘Threats, climate, outbreaks, and preparedness’; and ‘Harnessing innovation, technology, and artificial intelligence (AI)’. This theme and its associated subthemes were developed by the planning committee through collating responses for theme ideas sent as a survey to registrants of CCGH 2024 and through collaborative discussion. The selected topics invited policymakers, researchers, practitioners, community leaders and representatives, trainees, funders, and advocates to chart a path forward to sustainability, resiliency, and solutions that leaves no one behind.

The CCGH 2025 call for abstracts opened on 3 March 2025, with an overwhelming response of over 520 abstracts submitted from around the world. The conference ultimately convened 768 participants from 64 countries, with strong representation from students and emerging professionals (n = 168) and academics/researchers (n = 188). Low- and middle-income country (LMIC) participation was also strong, with attendees from Nigeria (n = 90), Haiti (n = 40), Guinea (n = 35), Ghana (n = 30), and Uganda (n = 23). The programme featured 15 symposia, 11 workshops, 245 oral presentations, 150 posters, and curated networking events (see **Online Supplementary Document** for the programme schedule). Building on the momentum from past conferences, such as those in 2023 [1] and 2024 [2], the CCGH 2025 further strengthened Canada’s position as a leading convenor of global health dialogue, equity-driven programming, and meaningful partnerships between LMICs and Canadian institutions.

On the closing day of the CCGH 2025, a dedicated workshop session brought together participants to identify future research directions. The session was organised into four breakout discussion groups, aligning with the conference’s four subthemes. In each group, participants were invited to reflect on significant drivers of change emerging from sessions they attended, identify gaps and/or opportunities for research, and reflect on new directions (**Box 1**). High-level summaries of these workshop questions and discussions are presented below.

Box 1 **Questions presented to workshop participants to reflect on the sub-themes**

Question 1: Reflect on the presentations you attended in this thematic stream and list up to three notable drivers of change impacting global health.

Question 2: Thinking about the presentations in this theme, what future research question or topic would you consider important to pursue?

Question 3: Thinking about your research, did the presentations generate new ideas that you hope to pursue? If so, please state the topic or question.

## SUBTHEME 1: ‘NAVIGATING GLOBAL HEALTH GOVERNANCE, FINANCING, COLLABORATION, AND TRUST’

Discussions on this conference subtheme narrowed in on inequities in global health, while addressing innovation, power dynamics, and local and international partnerships. For instance, this included highlighting the need for innovation in healthcare access, resources, and financing, which was considered alongside the implications of shifting power dynamics and leveraging public-private partnerships. Attendees also discussed approaches to integrate Indigenous knowledge, people-centred approaches, local leadership, and diplomacy into health policies and solutions. Finally, discussions explored approaches to enhance south-south and north-south collaborations in global health policy.

The conversation on global health governance was centred on difficult realities such as reduced funding, the emergence of harmful ideologies, and rising nationalism. An important question posed was ‘how will policymakers decide what to prioritise when resources are shrinking?’ Participants also noted challenges around lack of trust and multilateralism, as well as rapid technological changes creating new complexities for governance structures.

Participants noted a sharp drop in funding and resources, particularly from member countries of the Organisation for Economic Co-operation and Development, which is forcing a fundamental rethink around global health financing. Some individuals questioned whether definitions of high-income countries should be reconsidered based on power dynamics and capacity rather than just gross national income and called for marrying global health governance strategies with locally-driven development approaches. Participants also emphasised the need for localised, co-created solutions that are sustainable and cost-efficient. Additional themes included: building evidence on values and ideologies in global health governance; rethinking institutional decision-making structures; and examining Canada’s redefined role in global health leadership. See **Box 2** for questions raised by participants related to research gaps in global health governance.

Box 2 **Questions raised by participants related to research gaps in global health governance**

− How does technical expertise influence global and public health policy, and how can engagement between experts and policymakers be improved?
− Who defines 'local' and 'community' in global and public health governance, and to what extent do these definitions shape how community representation is conceptualised and put into practice?
− How can health governance frameworks ensure that the priorities of various populations, localities and communities are meaningfully reflected in higher-level (national) decision-making?
− What frameworks can guide equitable and sustainable resource allocation for countries unable to sustainably achieve domestic health financing?
− How do governments allocate health resources and expenditure while ensuring accountability?
− What enables civil society organisations to maintain independence and effectively represent community interests in health governance?
− Who has been guiding the way that global actors (*e.g.* World Health Organization; Gavi, the Global Financing Facility (hosted by the World Bank)) have been prioritising and how?

## SUBTHEME 2: ‘YOUTH, GENDER, AND INCLUSIVE LEADERSHIP’

This subtheme’s sessions and discussions explored the role of youth and gender in inclusive leadership. Conversations centred around the importance of empowering youth as change agents in global health, while addressing gender disparities and promoting gender-responsive health policies and solutions. The subtheme also emphasised the need to encourage inclusive leadership to foster diverse perspectives and solutions and strengthen mentorship, research collaboration, and practice for early-career professionals.

Participants emphasised the need to move towards co-creation models where youth are partners and drivers of change as opposed to beneficiaries. In other words, they highlighted that youth initiatives must be led by young people themselves, while considering their intersectional identities (*i.e.* gender, disability, and other factors). Key items included: participatory approaches; making sure youth are paid living wages; and scaling up work with civil society organisations.

Collaboration between people, institutions, and community-led centred partnerships was likewise emphasised. Several participants highlighted tension between global funding agendas and local priorities, noting that funding opportunities often force organisations to modify their projects to fit trends rather than specific needs within communities. Participants called for focusing on issues rather than places, and for support for those who are driving change, such as Indigenous youth.

In terms of future research, participants wanted to see evaluations of the effectiveness and economic impact of engaging youth leadership, including cost-benefit analyses to demonstrate returns on investment to policymakers. They also stated there is a need to engage more youth in multisectoral policy and to hold decision-makers accountable. Some questions arose about engaging youth in humanitarian settings and underserved communities, and how to sustain meaningful youth participation while maintaining a gender transformative lens.

A recurring theme was to go beyond just sexual and reproductive health and think about housing, employment, and food security, among other factors (*i.e.* all the interconnected social determinants of health). Climate change and health was also raised as an emerging priority. Participants called for funders to be more flexible within their mandates. Methodological innovations were also discussed, including moving beyond surveys on knowledge, attitudes, and practices to qualitative and arts-based methods for addressing stigmatised topics and engaging populations with differing abilities and literacy levels. Participants emphasised decolonising research approaches and using participatory action research methods designed for youth, by youth.

## SUBTHEME 3: ‘GLOBAL HEALTH SECURITY – THREATS, CLIMATE, OUTBREAKS, AND PREPAREDNESS’

The discussions under this subtheme focused on strengthening global health systems to prevent and respond to misinformation, pandemics, climate change, and planetary health. One dominant conclusion that emerged was a need for innovations in surveillance, early warning systems, and global collaboration, while responding to the health needs of populations in conflict zones and displaced communities. Discussions also underscored the need to advocate for health as a tool for peacebuilding and stability.

Participants noted a confluence of factors exacerbating the polycrises faced within global health. Discussions centred on unprecedented geopolitical instability that makes collective and coordinated action on global health threats extremely difficult. Participants highlighted that conflict zones face disrupted health systems and limited humanitarian access, while political tensions undermine cross-border coordination.

Climate change was identified as a major health threat multiplier, worsening food and water insecurity, human displacement, and the spread and re-emergence of previously controlled infectious diseases. Participants noted rising concerns about zoonotic spillover and antimicrobial resistance, alongside recent measles and other vaccine-preventable disease outbreaks that reveal gaps in coverage and preparedness; distrust in science and misinformation, and the role this is having in the emergence and re-emergence of diseases globally; and how do we, as health professionals (researchers, policymakers, *etc*.) work to engage with these groups to start an open dialogue, rather than just dismiss them. These overlapping challenges were seen as prohibitive for taking collective action on global health threats.

Participants also emphasised persistent inequities in preparedness, whereby LMICs continue to face gaps in surveillance, response capacity, training, and funding when compared with high-income countries. Concerns were raised that if such weaknesses are not addressed, collective global health security worldwide will continue to be undermined.

Regarding technology, participants acknowledged that while rapid changes in digital developments offer new possibilities, they are also reshaping the rules of global health security. Emerging tools like AI and digital surveillance were seen as offering potential for detection and response, but participants stressed that ethical, governance, and equity considerations are subsequently raised. For example, participants discussed the data that is being used to inform AI, including how these data are being used, ownership over the data collected, and if and/or how the data are being made publicly available, asking questions such as ‘are they relevant for the communities?’ and ‘do they reflect communities?’ There was strong emphasis on ensuring that LMICs and local communities are meaningfully included in discussions around implementation of these tools (including equitable access to such tools).

Discussions also highlighted declining governance and funding. Looking at overlapping crises with fragile coordination, participants noted that trust, coordination, and collective action are undermined in the context of overlapping crises. An important emerging concern raised was declining or unstable funding, including reductions from major donors. Participants expressed concerns that relying on a small number of funders exposes key programs to risk. Addressing these governance and funding challenges was identified as critical to building more resilient global health systems. See **Box 3** for questions raised by participants related to research gaps in global health security.

Box 3 **Questions raised by participants related to research gaps in global health security**

− How can health systems in LMICs be strengthened to anticipate and respond to climate-driven disease risks?
− How can community-based approaches improve early warning and rapid responses for emerging or re-emerging diseases?
− Considering that some countries may never fully shift to using their own resources, how should funders and the World Health Organization target their support and what frameworks should guide these choices?
− How can research inform engagement with political actors to ensure public health evidence translates into public policy action?
− How can digital surveillance tools and AI be used responsibly to improve outbreak detection while addressing ethical, governance, and equity concerns?
− What frameworks ensure LMICs and local communities are meaningfully included in decisions about technology adoption and use?
− How can locally-led governance be strengthened, ensuring that the priorities of communities served are reflected?

## SUBTHEME 4: ‘INNOVATION, TECHNOLOGY, AND AI’

Discussions within this subtheme focused on harnessing innovation, technology, and AI for global health. Sessions explored methods to scale-up frugal innovations for remote and resource-limited settings, while emphasising the need to explore the potential for AI, telemedicine, and data-driven science in healthcare delivery. How to bridge the digital divide to ensure equitable access to health technologies and examining innovative, sustainable financing models to future proof global health initiatives was also examined.

An important distinction was made regarding the semantics of innovation: innovation is not just technology or gadgets; programmes can be innovative, too. Participants noted that there was a need for better long-term impact evaluations, especially for capacity building and sustainability. Notably, tracking and disseminating outcomes at short-, intermediate-, and long-term intervals, rather than just presenting immediate results.

Participants discussed how innovation should come from local contexts, rather than having solutions imported to fit different cultures and resources. Furthermore, localisation emerged as a dominant theme throughout presentations, with strong emphasis on youth empowerment, particularly with the African continent, as this can lead innovation efforts. Several participants highlighted frugal innovation approaches and the importance of respecting community culture and needs from the outset.

As expected, AI was a major discussion topic, with calls for capacity building for AI leadership in Africa and rural/remote settings, education on the responsibleand ethical use of AI, as well as the need for governance frameworks and policies. Participants questioned how emerging technologies can improve healthcare delivery ethically while addressing the social determinants of health. The importance of monitoring and evaluating AI tools to determine effectiveness rather than promoting a digital divide, whereby technology adds to healthcare worker burden, was also raised. Further, the impacts of climate change on health security, the notion of ‘pilotitis’ (*i.e.* moving beyond pilots to sustainability), ensuring equitable access to innovations, harmonisation and cross-pollination of models that work and addressing the workforce capacity crisis, and rising burnout levels were also discussed. In conclusion, participants stressed that implementation should simplify healthcare workers’ workloads.

## CONCLUSIONS

The CCGH 2025 was held at a pivotal time for global health, given reduced global funding and associated changing climate for the subject matter. The host organisation, the Canadian Association for Global Health (CAGH), felt it was incredibly important to persist and host these important conversations at such a time. The CAGH prides itself as being a convener, partner, and champion for global health equity, and feels strongly that the conference plays a critical role in supporting conversations, networking, critiques, and mobilisation.

Discussions covered themes of global health governance, youth and gender leadership, health security, and innovation. In these conversations, participants emphasised the urgent need for locally-led solutions, meaningful community participation, and addressing persistent challenges (*e.g.* reduced funding, governance fragmentation, and climate change as a health threat multiplier). The discussions also underscored calls to decolonise global health research systems by centring voices from the global south and ensuring research agendas reflect community priorities rather than donor-driven trends.

Building on the successes of CCGH 2024, the 2025 conference continued implementing an equity-centred scholarship framework to encourage participation from global south attendees. As described by Gauhar *et al*. [3], this system used a six-step process for the creation of the scholarship, as well as considerations for recipient selection, methods for application scoring, and processes for scholarship distribution. This targeted funding method continued in 2025 and provides a foundation for future CCGHs and can be scaled up for other international conferences to promote inclusivity and representation. These efforts reflect the CAGH’s ongoing commitment to ensuring that global health dialogue includes voices affected by contemporary global health challenges.

Planning for CCGH 2026 is well under way; the conference is to be held in Montreal, Canada, on the traditional and unceded territory of the *Kanien’kehá:ka* (Mohawk) people, from 16 to 18 October 2026. Its theme will be: ‘Collectively reclaiming global health equity: Restoring commitment to action in a fragmented and uncertain world’. The subthemes to be explored are: ‘Addressing upstream determinants of health equity’; ‘Equity in action: health systems, resilient systems and communities, and centring marginalised voices’; and ‘From evidence to impact: practical approaches and tools for implementation and accountability’. We invite you to join us, whether in-person or virtually, at our annual conference.

## Additional material


Online Supplementary Document
