# Supplementary material for: Navigating the path forward: advancing global health in a changing world – the 31st Canadian Conference on Global Health in 2025
Source: J Glob Health. 2026 Jul 3;16:02001. doi: 10.7189/jogh.16.02001 (PMC13329881; doi:10.7189/jogh.16.02001)
Supplement: Online Supplementary Document [file jogh-16-02001-s001.pdf]

Supplement to: Mutumba-Nakalembe MJ, O'Hearn S, Manga JC, Soverall C, Khorramizadeh R, Kruja K, MacDonald NE, Mac-Seing M, Sohani S, Tiedje KE, Enright K, Khaled K, Macrae K, Barimah KB, Roberts JH, Davison CM, Hassan S, Amri M. Navigating the path forward: advancing global health in a changing world – the 31st Canadian Conference on Global Health in 2025. J Glob Health. 2026;16:02001.

Summary Program Schedule

Friday, October 24

WELCOME & OPENING REMARKS
8:00 AM - 8:30 AM (30 minutes)

OPENING KEYNOTE: NAVIGATING THE DIGITAL HEALTH FRONTIER
8:30 AM - 9:00 AM (30 minutes)

VIRTUAL SYMPOSIUM 1: Centering communities, driving change: Co-creating family strengthening programs to improve global health
9:00 AM - 10:30 AM (90 minutes)

VIRTUAL SYMPOSIUM 2: Climate Hub: Providing Humanitarian Aid in Climate Emergencies
9:00 AM - 10:30 AM (90 minutes)

VIRTUAL SYMPOSIUM 3: Health and social protection of women workers in the care economy: Experiences from Argentina, Ecuador and Peru
9:00 AM - 10:30 AM (90 minutes)

VIRTUAL SYMPOSIUM 4: Building Cultural Humility, Advocacy and Professional Competencies of Future Global Health Leaders through Structured Experiential Learning Courses
9:00 AM - 10:30 AM (90 minutes)

NETWORKING BREAK
10:30 AM - 11:00 AM (30 minutes)

VIRTUAL SYMPOSIUM 5: From research to resilience: Advancing health and economic well-being for the youth in the informal sector in East and West Africa
11:00 AM - 12:30 PM (90 minutes)

VIRTUAL SYMPOSIUM 6: Integrating climate action into community health programming to ensure better health outcomes, security, and system resilience in Africa
11:00 AM - 12:30 PM (90 minutes)

VIRTUAL WORKSHOP 1: Hacking Digital Health: From Ideas to Impact in Tech & Entrepreneurship
11:00 AM - 12:30 PM (90 minutes)

VIRTUAL WORKSHOP 2: Transforming adolescent sexual reproductive health in Pakistan: a holistic and culturally sensitive strategy
11:00 AM - 12:30 PM (90 minutes)

LUNCH BREAK & NETWORKING
12:30PM - 1:00 PM (30 minutes)

VIRTUAL ORAL 1: Global Health Security: Threats, Climate, Outbreaks and Preparedness
1:00 PM - 3:00 PM 120 minutes)

VIRTUAL ORAL 2: Navigating Governance, Financing, Collaboration and Trust in Global Health
1:00 PM - 3:00 PM 120 minutes)

VIRTUAL ORAL 3: Youth, Gender, and Inclusive Leadership in Global Health
1:00 PM - 3:00 PM 120 minutes)

VIRTUAL ORAL 4: Innovation, Technology and Artificial Intelligence for Global Health
1:00 PM - 3:00 PM 120 minutes)

NETWORKING BREAK
3:00 PM - 3:15 PM (15 minutes)

VIRTUAL POSTER PRESENTATIONS
3:15 PM - 4:45 PM (30 minutes)

CLOSING REMARKS
4:45 PM - 5:00 PM (30 minutes)

CCGH AFTER HOURS
6:00 PM - 9:00 PM

Saturday, October 25

STUDENTS & EMERGING PROFESSIONALS PROGRAM

WELCOME & OPENING REMARKS
10:00 AM - 10:15 AM (15 minutes)

PLENARY PANEL: TRENDS IN GLOBAL HEALTH
10:15 AM - 11:15 AM (60 minutes)

NETWORKING BREAK
11:15 AM - 11:30 AM (15 minutes)

NETWORKING MATCH & MINGLE
11:30 AM - 12:30 PM (60 minutes)

NETWORKING LUNCH
12:30 PM - 1:30 PM (60 minutes)

GETTING HIRED IN GLOBAL HEALTH: RESUME AND JOB SEARCH ESSENTIALS
1:30 PM - 3:45 PM (135 minutes)

VACCINATING THE FUTURE: A WORKATHON
1:30 PM - 3:45 PM (135 minutes)

WALK & TALK: PATHWAYS TO CONNECTIONS
1:30 PM - 3:45 PM (135 minutes)

HISTORICAL HALIFAX WALKING TOUR
2:00 PM - 4:00 PM (120 minutes)

———

VOLUNTEER: ADSUM HOUSE FOR WOMEN & CHILDREN
2:15 PM - 4:30 PM (135 minutes)

COFFEE & NETWORKING BREAK
3:45 PM - 4:00 PM (15 minutes)

GLOBAL HEALTH QUIZ
4:00 PM - 5:00 PM (60 minutes)

VACCINATING THE FUTURE: A WORKATHON
4:00 PM - 5:00 PM (60 minutes)

CLOSING REMARKS
5:00 PM - 5:15 PM (15 minutes)

SEP DINNER
5:30 PM - 7:30 PM

CCGH AFTER HOURS
6:00 PM - 9:00 PM

08:00 08:05 08:10 08:15 08:20 08:25 08:30 08:35 08:40 08:45 08:50 08:55 09:00 09:05 09:10 09:15 09:20 09:25 09:30 09:35 09:40 09:45 09:50 09:55 10:00 10:05 10:10 10:15 10:20 10:25 10:30 10:35 10:40 10:45 10:50 10:55 11:00 11:05 11:10 11:15 11:20 11:25 11:30 11:35 11:40 11:45 11:50 11:55 12:00 12:05 12:10 12:15 12:20 12:25 12:30 12:35 12:40 12:45 12:50 12:55 13:00 13:05 13:10 13:15 13:20 13:25 13:30 13:35 13:40 13:45 13:50 13:55 14:00 14:05 14:10 14:15 14:20 14:25 14:30 14:35 14:40 14:45 14:50 14:55 15:00 15:05 15:10 15:15 15:20 15:25 15:30 15:35 15:40 15:45 15:50 15:55 16:00 16:05 16:10 16:15 16:20 16:25 16:30 16:35 16:40 16:45 16:50 16:55 17:00 17:05 17:10 17:15 17:20 17:25 17:30 17:35 17:40 17:45 17:50 17:55 18:00 18:05 18:10 18:15 18:20 16:25 18:30 18:35 18:40 18:45 18:50 18:55

Sunday, October 26

REGISTRATION
8:00 AM - 6:00 PM

ORAL PRESENTATION 1: Global Health Equity at the Intersection of Migration, Environment and Social Determinants
8:00 AM - 9:30 AM (90 minutes)

ORAL PRESENTATION 2: Social and structural determinants of health among vulnerable populations
8:00 AM - 9:30 AM (90 minutes)

ORAL PRESENTATION 3: Interconnected Crises: Climate Change, Governance, and Emerging Threats
8:00 AM - 9:30 AM (90 minutes)

ORAL PRESENTATION 4: Smart Systems & Surveillance: Strengthening Health Information with AI & Digital Tools
8:00 AM - 9:30 AM (90 minutes)

NETWORKING BREAK
9:30 AM - 10:00 AM (30 minutes)

ORAL PRESENTATION 5: Transforming Global Health Systems: Addressing social, environmental and governance challenges through policy and implementation
10:00 AM - 11:30 AM (90 minutes)

ORAL PRESENTATION 6: Transforming sexual and reproductive health through evidence and action
10:00 AM - 11:30 AM (90 minutes)

ORAL PRESENTATION 7: Pandemics, Prevention, and Vaccine Equity: Strengthening Global Health Security
10:00 AM - 11:30 AM (90 minutes)

ORAL PRESENTATION 8: Scanning the Future: Reviews & Reflections on AI, Innovation, and Global Health Trends
10:00 AM - 11:30 AM (90 minutes)

NETWORKING LUNCH
11:30 AM - 12:30 PM (90 minutes)

CCGH 2025 OPENING CEREMONY / CCSM 2025 : CÉRÉMONIE D'OUVERTURE
PLENARY #1: NAVIGATING GOVERNANCE, FINANCING, COLLABORATION & TRUST IN GLOBAL HEALTH
12:30 PM - 2:30 PM (120 minutes)

SYMPOSIA 1: Advancing Global Health Preparedness: Collaborative Approaches to Develop Mobile Health Teams in Crisis Settings
2:30 PM - 4:00 PM (90 minutes)

SYMPOSIA 2: Rethinking Global Health Education – Leadership, Pedagogy & Possibilities
2:30 PM - 4:00 PM (90 minutes)

WORKSHOP 1: Development, evaluation, and analysis of Knowledge, Attitudes and Practice (KAP) questionnaires: From internal consistency to the behavior theory
2:30 PM - 4:00 PM (90 minutes)

WORKSHOP 5: Strengthening Health Systems in LMIC Through Responsible AI
2:30 PM - 4:00 PM (90 minutes)

ORAL PRESENTATION 9: Power, Politics & Policy: Who Really Shapes Global Health
4:00 PM - 5:00 PM (60 minutes)

ORAL PRESENTATION 10: Community voices and Participatory Health Research in Global Health
4:00 PM - 5:00 PM (60 minutes)

ORAL PRESENTATION 11: Mental Health, Displacement, and the Humanitarian Imperative
4:00 PM - 5:00 PM (60 minutes)

ORAL PRESENTATION 12: Bridging Gaps in Crisis: Digital Health Solutions for Vulnerable Populations
4:00 PM - 5:00 PM (60 minutes)

POSTER VIEWING & NETWORKING
5:00 PM - 6:00 PM (60 minutes)

CAGH AWARDS CEREMONY & WELCOME RECEPTION
6:00 PM - 7:30 PM (90 minutes)

Monday, October 27

REGISTRATION / EXHIBITS
8:00 AM - 4:00 PM

NETWORKING BREAKFAST
8:00 AM - 9:00 AM (60 minutes)

PLENARY #2: HARNESSING INNOVATION: RESULTS FOR WHOM, BY WHOM?
9:00 AM - 10:30 AM (90 minutes)

NETWORKING BREAK / EXHIBITS / POSTERS VIEWING
10:30 AM - 11:00 AM (30 minutes)

SYMPOSIA 3: Strengthening the frontline globally and in Canada: a call for economic and gender justice for community-based health workers
11:00 AM - 12:30 PM (90 minutes)

SYMPOSIA 4: Adolescent and Indigenous knowledge and beliefs in sexual and reproductive health and mental health research: Experiences from Guatemala, Mexico and Peru
11:00 AM - 12:30 PM (90 minutes)

WORKSHOP 3: Creating meaningful community, government and multinational academic partnerships to decolonize Global Health Research
11:00 AM - 12:30 PM (90 minutes)

WORKSHOP 4: Leveraging the Power of Civil Society: Building Trust and Resiliency in Sexual and Reproductive Health and Rights
11:00 AM - 12:30 PM (90 minutes)

NETWORKING LUNCH / CAGH ANNUAL GENERAL MEETING / EXHIBITS / POSTERS VIEWING
12:30 PM - 2:00 PM (90 minutes)

SYMPOSIA 5: Preparing for the next pandemic: lessons from viral hemorrhagic fevers & the evolution of Safe and Dignified Burials in the Red Cross Red Crescent Movement
2:00 PM - 3:30 PM (90 minutes)

SYMPOSIA 6: Protecting our Collective Future: Renewing Canada's Role in Global Health
2:00 PM - 3:30 PM (90 minutes)

SYMPOSIA 7: Artificial Intelligence and Global Health: Predicting Vulnerability in the Grassroots waste pickers, Ecuador
2:00 PM - 3:30 PM (90 minutes)

WORKSHOP 6: World Vision Canada's Urban AHADI SRHR Tanzania Project: Youth Empowerment, Gender Equality and Inclusive Leadership
2:00 PM - 3:30 PM (90 minutes)

NETWORKING BREAK / EXHIBITS / POSTERS VIEWING
3:30 PM - 4:00 PM (30 minutes)

ORAL PRESENTATION 13: Beyond the Budget: Gender, Generations & the Politics of Health Funding
4:00 PM - 5:00 PM (60 minutes)

ORAL PRESENTATION 14: Gender-inclusive strategies for health equity and empowerment
4:00 PM - 5:00 PM (60 minutes)

ORAL PRESENTATION 15: Infectious Disease Surveillance and the Power of Data: New Evidence and Enduring Threats
4:00 PM - 5:00 PM (60 minutes)

ORAL PRESENTATION 16: MedTech for Impact: Frugal and High-Tech Solutions to Global Health Challenges
4:00 PM - 5:00 PM (60 minutes)

POSTER VIEWING & NETWORKING
5:00 PM - 6:00 PM (60 minutes)

CCGH AFTER HOURS
6:00 PM - 9:00 PM
PARTNER NETWORKING EVENTS: WOMEN IN GLOBAL HEALTH

Time	Tuesday, October 28			
08:00	**NETWORKING BREAKFAST**<br>8:00 AM - 8:45 AM (45 minutes)			
08:05				
08:10				
08:15				
08:20				
08:25				
08:30				
08:35				
08:40				
08:45	**PLENARY #3:** GLOBAL HEALTH AT A CROSSROADS<br>8:45 AM - 10:30 AM (90 minutes)			
08:50				
08:55				
09:00				
09:05				
09:10				
09:15				
09:20				
09:25				
09:30				
09:35				
09:40				
09:45				
09:50				
09:55				
10:00				
10:05				
10:10				
10:15				
10:20				
10:25				
10:30	**NETWORKING BREAK**<br>10:30 AM - 10:45 AM (15 minutes)			
10:35				
10:40				
10:45	**WORKSHOP 7:**<br>Future Research Directions in a Changing World<br>10:45 AM - 12:15 PM<br>(90 minutes)	**SYMPOSIA 8:**<br>Fragile Settings, Unbreakable Resolve: Lifesaving Nutrition Services in Conflict and Chaos<br>10:45 AM - 12:15 PM<br>(90 minutes)		**WORKSHOP 8:**<br>Leadership féminin et santé : Les CFU, un modèle d'innovation sociale à explorer<br>10:30 AM - 12:00 PM<br>(90 minutes)
10:50				
10:55				
11:00				
11:05				
11:10				
11:15				
11:20				
11:25				
11:30				
11:35				
11:40				
11:45				
11:50				
11:55				
12:00				
12:05				
12:10				
12:15	**ORAL PRESENTATION 17:**<br>From Global Policy to Local Capacity: Innovation in Health system support and patient engagement<br>12:15 PM - 1:15 PM<br>(60 minutes)	**ORAL PRESENTATION 18:**<br>Redefining leadership: gender, equity and inclusion in global health<br>12:15 PM - 1:15 PM<br>(60 minutes)	**ORAL PRESENTATION 19:**<br>Sexual and Reproductive Health Rights in the Context of Crisis and Change<br>12:15 PM - 1:15 PM<br>(60 minutes)	**ORAL PRESENTATION 20:**<br>AI, Big Data & Predictive Health in Global Contexts<br>12:15 PM - 1:15 PM<br>(60 minutes)
12:20				
12:25				
12:30				
12:35				
12:40				
12:45				
12:50				
12:55				
13:00				
13:05				
13:10				
13:15	**CLOSING LUNCH & CCGH 2025 AWARDS**<br>1:15PM - 2:15 PM (60 minutes)			
13:20				
13:25				
13:30				
13:35				
13:40				
13:45				
13:50				
13:55				
14:00				
14:05				
14:10				
14:15	**ORAL PRESENTATION 21:**<br>Building Trust and Inclusion in Global and Local Health Governance<br>2:15 PM - 3:15 PM<br>(60 minutes)	**ORAL PRESENTATION 22:**<br>Youth empowerment strategies for health, equity and change<br>2:15 PM - 3:15 PM<br>(60 minutes)	**ORAL PRESENTATION 23:**<br>Outbreaks and Operational Readiness: Responding to Epidemics and Strengthening Surveillance<br>2:15 PM - 3:15 PM<br>(60 minutes)	**ORAL PRESENTATION 24:**<br>Inclusive Digital Care: Adherence, and Equity in Maternal, Adolescent, SRH and Community Health<br>2:15 PM - 3:15 PM<br>(60 minutes)
14:20				
14:25				
14:30				
14:35				
14:40				
14:45				
14:50				
14:55				
15:00				
15:05				
15:10				
15:15	**ORAL PRESENTATION 25:**<br>Advancing Equity Through Health Governance: Global Perspectives, Local Impact<br>3:15 PM - 4:15 PM<br>(60 minutes)	**ORAL PRESENTATION 26:**<br>Rethinking Youth Participation, Influence, and Leadership<br>3:15 PM - 4:15 PM<br>(60 minutes)	**ORAL PRESENTATION 27:**<br>Frontline Realities: Women's Health, Workforce Safety, and Equity in Challenging Contexts<br>3:15 PM - 4:15 PM<br>(60 minutes)	**ORAL PRESENTATION 28:**<br>Educating for Change: Digital Tools in Health Literacy, SRHR, and Capacity Building<br>3:15 PM - 4:15 PM<br>(60 minutes)
15:20				
15:25				
15:30				
15:35				
15:40				
15:45				
15:50				
15:55				
16:00				
16:05				
16:10				
16:15				
16:20				
16:25				
16:30				
16:35				
16:40				
16:45				
16:50				
16:55				
17:00				
17:05				
17:10				
17:15				
17:20				
17:25				
17:30				
17:35				
17:40				
17:45				
17:50				
17:55				
18:00	**CCGH AFTER HOURS**<br>6:00 PM - 9:00 PM			
18:05				
18:10				
18:15				
18:20				
16:25				
18:30				
18:35				
18:40				
18:45				
18:50				
18:55				

Detailed Program Schedule

Day 1 (Virtual Day) - Friday, October 24, 2025

**8:00 a.m.**	**WELCOME & OPENING REMARKS**
8:00 a.m.	WELCOME TO CCGH 2025 & LAND ACKNOWLEDGEMENT
8:05 a.m.	CCGH 2025 CONFERENCE CO-CHAIR REMARKS
8:10 a.m.	REMARKS ON BEHALF OF CAGH: MEMBERSHIP, THANK VOLUNTEERS AND PARTNERS, AND INTRODUCE THE MINISTER'S VIDEO MESSAGE
8:15 a.m.	**VIDEO:** REMARKS FROM GLOBAL AFFAIRS CANADA
8:19 a.m.	RESPONSE TO MINISTER'S REMARKS
8:21 a.m.	HOUSEKEEPING ANNOUNCEMENTS
**8:30 a.m.**	**OPENING KEYNOTE**
8:30 a.m.	INTRODUCE OPENING KEYNOTE
8:35 a.m.	OPENING KEYNOTE: NAVIGATING THE DIGITAL HEALTH FRONTIER
8:50 a.m.	MODERATED Q&A SESSION FROM VIRTUAL ATTENDEES
9:00 a.m.	WRAP-UP & CLOSING REMARKS
**9:00 a.m.**	**CONCURRENT SYMPOSIA SESSIONS**
9:00 a.m.	VIRTUAL SYMPOSIUM 1: Centering communities, driving change: Co-creating family strengthening programs to improve global health
9:00 a.m.	VIRTUAL SYMPOSIUM 2: Climate Hub: Providing Humanitarian Aid in Climate Emergencies
9:00 a.m.	VIRTUAL SYMPOSIUM 3: Health and social protection of women workers in the care economy: Experiences from Argentina, Ecuador and Peru
9:00 a.m.	VIRTUAL SYMPOSIUM 4: Building Cultural Humility, Advocacy and Professional Competencies of Future Global Health Leaders through Structured Experiential Learning Courses
**10:30 a.m.**	**NETWORKING BREAK**
9:30 a.m.	NETWORKING ACTIVITY: Breakout rooms with conversation starters
**11:00 a.m.**	**CONCURRENT SYMPOSIA & WORKSHOPS**
11:00 a.m.	VIRTUAL SYMPOSIUM 5: From research to resilience: Advancing health and economic well-being for the youth in the informal sector in East and West Africa
11:00 a.m.	VIRTUAL SYMPOSIUM 6: Integrating climate action into community health programming to ensure better health outcomes, security, and system resilience in Africa
11:00 a.m.	VIRTUAL WORKSHOP 1: Hacking Digital Health: From Ideas to Impact in Tech &

	Entrepreneurship
11:00 a.m.	VIRTUAL WORKSHOP 2: Transforming adolescent sexual reproductive health in Pakistan: a holistic and culturally sensitive strategy
**12:30 p.m.**	**LUNCH BREAK**
12:30 a.m.	NETWORKING ACTIVITY: Trivia/Quiz
**1:00 p.m.**	**CONCURRENT ORAL SESSIONS**
**1:00 p.m.**	**VIRTUAL ORAL 1: GLOBAL HEALTH SECURITY: THREATS, CLIMATE, OUTBREAKS AND PREPAREDNESS**
1:05 p.m.	Determinants of adherence to malaria preventive services among HIV-positive antenatal clinic attendees in Abuja Municipal Area Council (AMAC), FCT, Nigeria
1:13 p.m.	Antecedents and consequences of workplace violence against nurses in the context of Pakistan
1:21 p.m.	Feasibility of Implementing a Workplace Violence Reporting System in the Context of Pakistan
1:29 p.m.	Challenges and Mitigation Strategies for Affordable Medical Oxygen Provision for Mothers and Children in Conflict-Affected Regions of Northern Ethiopia
1:37 p.m.	Le concept de capacité adaptative au changement climatique est-il pertinent pour la santé publique ? Le cas des petits États insulaires caribéens
1:45 p.m.	Assessment of Integration of the COVID-19 Package in Primary Health Care and Routine Immunization in the DRC: Lessons Learned and Recommendations
1:53 p.m.	The effects of orphanhood and lack of parental care on child vaccination: Analyses of 189 cross-sectional household surveys from 82 low- and middle-income countries, 2005 to 2022
2:01 p.m.	Réponses endogènes à la COVID-19 en contexte de ressources limitées : étude de cas à l'Hôpital Général de Référence de Niamey
2:09 p.m.	Role of viral clade, GDP per capita and population density in the 2024 mpox epidemic in Africa: A retrospective cross-country study
2:17 p.m.	Enhancing Adherence and Minimizing Lost to Follow-Up: The Role of an Innovative Patient Advisory Board for Individuals with Type 1 Diabetes in Maryland County, Liberia - A Three-Year Analytical and Feasibility Study
2:25 p.m.	Opérationnalisation de l'approche « Une Santé » dans la surveillance des zoonoses dans un contexte de répartition inégale des ressources en République démocratique du Congo : Cas de la province de l'Equateur
2:33 p.m.	Unravelling the dynamics of Dengue in Oromia, Ethiopia, 2024
**1:00 p.m.**	**VIRTUAL ORAL 2: NAVIGATING GOVERNANCE, FINANCING, COLLABORATION AND TRUST IN GLOBAL HEALTH**
1:05 p.m.	Enacting Accountability: A Network Analysis of Global Health System and Purdue Pharmaceuticals

1:13 p.m.	Breastfeeding Experiences of African-Black Immigrant Women in Saskatoon, Saskatchewan: An Interpretative Phenomenological Approach
1:21 p.m.	Examining Bribery in the Pharmaceutical Sector: A Review of OECD Working Group Phase Reports
1:29 p.m.	Non-take-up of the Government of India's social protection schemes under Direct Benefit Transfer (DBT): A scoping review
1:37 p.m.	National Essential Medicines List Selection Process Design Effectiveness: Instrument Development and Testing
1:45 p.m.	Factors influencing primary care access for common mental health conditions among adults in West Africa: A scoping review
1:53 p.m.	Grounding McGill's dietetics education in Indigenous cultural safety: a pathway for truth and reconciliation in Canada (Thesis Proposal)
2:01 p.m.	Navigating the Path Forward: Advancing Global Health in a Changing World
2:09 p.m.	Aid Withdrawal or Wake-Up Call? Rethinking Health Sovereignty in Pakistan's Post-Donor Landscape
2:17 p.m.	A response to the impact of stop work order for continued HIV service delivery in Uganda. Insights from sentinel sites on HIV-self testing service delivery and reporting
2:25 p.m.	Multisectoral Collaboration for Universal Health Coverage in Africa: Governance Models and Implementation Experiences
2:33 p.m.	Répondre aux besoins locaux : vers une gouvernance plus souple des services à domicile
**1:00 p.m.**	**VIRTUAL ORAL 3: YOUTH, GENDER, AND INCLUSIVE LEADERSHIP IN GLOBAL HEALTH**
1:05 p.m.	Strengthening Food Culture through Participatory Action Research with Children in a Marginalized Area of Mexico City
1:13 p.m.	Harnessing leadership experiences for improved healthcare delivery: A scoping review of the experiences of healthcare managers and leaders in navigating crisis
1:21 p.m.	Bridging the Gap: Aligning Global SRH Policies with Local Realities in Malawi
1:29 p.m.	Youth Leadership Transforming Family Planning Access in Kenya, Nepal, and Zambia
1:37 p.m.	Gender-responsive adolescent sexual and reproductive health programming in West Africa: insights from a longitudinal qualitative implementation research study
1:45 p.m.	Empowering Youth: Enhancing Gender Equity and Health Equity Through the IMPACTO Project in Mozambique
1:53 p.m.	Élargir l'accès aux messages de planification familiale aux personnes vivant avec un handicap (personnes sourdes et malentendantes)
2:01 p.m.	Knowledge, Attitude and Practice on HIV/AIDS among University Students in Kilimanjaro Tanzania aged 18 to 45 years. A Cross-sectional Study
**1:00 p.m.**	**VIRTUAL ORAL 4: INNOVATION, TECHNOLOGY AND ARTIFICIAL INTELLIGENCE FOR GLOBAL HEALTH**
1:05 p.m.	EasyTract - A Table-Mounted Retractor for Low Resource Hospitals

1:13 p.m.	Assessment of Attitudes to Digital Health Technologies by Staff of Health Facilities in Fako Division of Cameroon
1:21 p.m.	Assessment of Intention to Use Digital Health Technologies by Staff of Health Facilities in Fako Division of Cameroon
1:29 p.m.	Pilot Testing and Validation of an AI-Based Mobile Application for Cervical Cancer Screening Using Visual Inspection with Acetic Acid (VIA) in Kigali, Rwanda
1:37 p.m.	Enhancing Quality of Health Care Delivered to Children in Kenya Through the Use of a mHealth Digital Platform
1:45 p.m.	In Vitro Antibacterial Activity of Honey against Clinical Isolates Associated with Urinary Tract Infections
1:53 p.m.	Leveraging AI for Safer Birth: NeMa smartbot integrated with the Safe Delivery App
2:01 p.m.	The 1000 Challenge: Facilitating Equity in Healthcare Research Leadership
2:09 p.m.	Enhancing Adherence and Minimizing Lost to Follow-Up: The Role of an Innovative Patient Advisory Board for Individuals with Type 1 Diabetes in Maryland County, Liberia - A Three-Year Analytical and Feasibility Study
2:17 p.m.	Harnessing Machine Learning to Predict Adverse Outcomes in Resource-Limited Settings: A Multi-Study Synthesis and Predictive Framework
2:25 p.m.	Effectiveness of an e-Health Application to Improve Iron Tablet Compliance Among Adolescent Girls: A Quasi-Experimental Study
2:33 p.m.	Delivering a hybrid dementia imaging training program in Africa
2:41 p.m.	Annexing Machine Learning (ML) in Antimicrobial Resistance Surveillance System in Sub-Saharan Africa: Feasibility, Scalability, and Recommendations
**3:00 p.m.**	**BREAK**
3:00 p.m.	NETWORKING ACTIVITY: Word Cloud Wrap-Up
**3:15 p.m.**	**POSTER PRESENTATIONS**
3:20 p.m.	Challenges in Global Distribution and Equitable Access to Monkeypox Vaccines
3:25 p.m.	Fathers' Perceptions of Competence in Kangaroo Mother Care (KMC): Toward Equitable Parental Participation and Trust in Neonatal Health Systems
3:30 p.m.	Réformes ascendantes et expertise contextuelle : réformer par le bas
3:35 p.m.	Institutional Facilitators and Barriers to Vaccine Access in Canada
3:40 p.m.	Assessing clinicians' awareness and perceptions of patients' rights in Mulago National Referral Hospital
3:45 p.m.	Bangladesh's Public Health Response at the Onset of the Global Mpox Outbreak in 2024
3:50 p.m.	Assessing the Integration of Climate Change into Public Health Policies in Africa: The Kingdon Multiple Streams Framework Analysis
3:55 p.m.	The impact of air pollution on health in Rwandan schools using polluting fuels for cooking

4:00 p.m.	COVID-19 Vaccine Booster Hesitancy Among University Students: Implications for Global Health Security and Pandemic Preparedness
4:05 p.m.	Automated Disinfection System in a Patient Diagnostic Trolley/Couch
4:10 p.m.	Mobile-Based Life Skills Education for Mental Health Among Internally Displaced Persons in Northern Nigeria: Exploring Adoption Factors and Service Delivery Implications
4:15 p.m.	A rapid review of the current and potential future use of drones to improve access to healthcare in Sub-Saharan Africa
4:20 p.m.	Climate Medicine Literacy Opportunities: AI Driven Risk Assessment Models for Medical Students
4:25 p.m.	Bridging the Knowledge GAP : Health care providers' Experiences,Insights and Awareness of Oral Cancer
4:30 p.m.	Climate Change Knowledge and Perception and associated habits in Sunamganj, Bangladesh
4:35 p.m.	"People say it kills…very fast…you get scared"- Experiences, Perspectives and Coping Mechanisms of people living with Hypertension and diabetes in Southern Ghana.
**4:45 p.m.**	**WRAP UP & CLOSING REMARKS**

Day 2 (SEP Day) - Saturday, October 25, 2025

Time	Stream 1	Stream 2	Stream 3
10:00–10:15	Welcome & SEP Bingo Launch		
10:15–11:15	Panel: Trends in Global Health		
11:15-11:30	Networking Break		
11:30-12:30	Networking Match Hour		
12:30–13:30	Networking Lunch		
13:30–15:45	Workathon: Solve This Case	Resume + LinkedIn Workshop	Walk'n'talk/city walk/speed dating
15:45-16:00	Networking Break		
16:00–17:00	Workathon: Solve This Case	Global Health Quiz: Bold Voices, Real Impact	
17:00-17:30	Closing		
17.30–19:00	SEP Dinner		

Day 3 - Sunday, October 26, 2025

**8:00 a.m.**	**CONCURRENT ORAL SESSIONS**
**8:00 a.m.**	**ORAL 1: GLOBAL HEALTH EQUITY AT THE INTERSECTION OF MIGRATION, ENVIRONMENT AND SOCIAL DETERMINANTS**
8:05 a.m.	Impact of Stress on the Psychosocial Well-being of Black African Migrant Parents Facing Transnational Family Separation in Hamilton+A17:A26
8:13 a.m.	Key Components of Effective Immigrant Settlement and Integration Organizations Supporting Immigrant Access and Utilization of Health-Related Services: A Scoping Review Protocol
8:21 a.m.	Why urban communities from low- and middle-income countries participate in global health research: A scoping review using fuzzy cognitive mapping
8:29 a.m.	Catalyzing Climate Adaptation for Healthcare Equity in Vulnerable Communities: Insights from the World's Largest Floating Slum
8:37 a.m.	Environmental Barriers and Bridges: How Built Spaces Impact Mental Health i[illegible] Racialized Immigrant Older Adults - A Qualitative Study
8:45 a.m.	Devolving Immigrant Recruitment and Settlement Services to the Local Level: An Explanatory Case Study of Immigrant Health and Social Services in Rural British Columbia
8:53 a.m.	Diagnosis, Associated Factors, and Morbimortality of Preterm Infants with Extrauterine Growth Restriction in Three Kangaroo Mother Care Programs in Colombia
9:01 a.m.	Between Borders and Bodies: Intersectional Adjudication in FGM-Related Refugee Claims in Canada
9:09 a.m.	Barriers and Facilitators to Engagement in Collective Gardening Among Black African Immigrants in Edmonton, Alberta
**8:00 a.m.**	**ORAL 2: SOCIAL AND STRUCTURAL DETERMINANTS OF HEALTH AMONG VULNERABLE POPULATIONS**
8:05 a.m.	Rural youth perspectives on planetary health and climate injustice: A Ghanaia[illegible] case study
8:13 a.m.	Intimate partner violence and women's nutrition: A multi-country analysis and implications for cross-sectoral programming
8:21 a.m.	Thinking Syndemically About Maternal-Child Health: A Focus on Nepal
8:29 a.m.	Gendered Lessons from Post-COVID-19 Recovery in Kenya's Informal Food Sector-A Case Study of Kisumu City

8:37 a.m.	Health risks, outcomes, and gendered responses of non-migrating household members amid widespread male out-migration in the Western Highlands of Guatemala
8:45 a.m.	Syndemics of Housing insecurity and HIV/AIDS: Effects on Dermatological Conditions among PLHIV in Kenya
8:53 a.m.	Where have all the mothers gone? COVID and mothers leaving the labor force in Egypt
9:01 a.m.	The Intersection of HIV and Mental Health: A Study of Depression Among Adolescents and Young Adults in Rwanda
9:09 a.m.	Factors Associated with Anxiety Among Adolescents and Young Adults Living with HIV in Rwanda: A Cross-Sectional Study
9:17 a.m.	Predictors Of Treatment Interruption Among Patients On Antiretroviral Therapy From An HIV Prevention Program In Eswatini.
8:00 a.m.	**ORAL 3: INTERCONNECTED CRISES: CLIMATE CHANGE, GOVERNANCE, AND EMERGING THREATS**
8:05 a.m.	Global health under a planetary emergency: Contending with waste and wastewater in Lusaka, Zambia
8:13 a.m.	Enhanced Situational Awareness: A Multi-Level Approach to Environmental Hazard Detection in Kampala Metropolitan Area
8:21 a.m.	Navigating Climate Threats to Global Health Security: Assessing the Impact of temperature and rainfall on Malaria Transmission in Rwanda (2012-2021)
8:29 a.m.	Health System Preparedness for Climate-Sensitive Vector-Borne Disease Outbreaks in Eastern Ethiopia: Gaps, Challenges, and Opportunities
8:37 a.m.	How inclusive is climate-related access to health services for people with disabilities and their animals? An exploratory study in Québec (Canada) and Gulu (Uganda)
8:45 a.m.	Malaria in the Era of Climate Change in Africa: A Rapid Evidence Synthesis
8:53 a.m.	Can indigenous knowledge systems explain the impact of climate change on pregnancy and childbirth? Oral stories from Ugandan communities
8:00 a.m.	**ORAL 4: SMART SYSTEMS & SURVEILLANCE: STRENGTHENING HEALTH INFORMATION WITH AI & DIGITAL TOOLS**
8:05 a.m.	A Smart Connected System for Integrated Health Administration
8:13 a.m.	SEEDNet: Enhancing Global Health Interventions with Settlement-Level Epidemiological Data
8:21 a.m.	From Fragmentation to Integration: Strengthening Digital Disease Surveillance in the ECOWAS Region

8:29 a.m.	Sijilli: A Cloud-Based Electronic Health Record for Refugees and Displaced Populations in Low Resource Settings
8:37 a.m.	Digitizing Maternal Health Records to Improve Continuity of Care in Rural Ethiopia: A Mixed-Methods Evaluation
8:45 a.m.	Predictive Modelling of Vaccination Dropout and Zero-Dose Status in India
8:53 a.m.	Piloting DHIS2 Android App For Family Planning Service Monitoring In Remote Areas With Low-Cadre Staff
9:30 a.m.	**NETWORKING BREAK**
9:30 a.m.	**NETWORKING ACTIVITY: Interest-Based Breakout Rooms**
10:00 a.m.	**CONCURRENT ORAL SESSIONS**
10:00 a.m.	**ORAL 5: TRANSFORMING GLOBAL HEALTH SYSTEMS: ADDRESSING SOCIAL, ENVIRONMENTAL AND GOVERNANCE CHALLENGES THROUGH POLICY AND IMPLEMENTATION**
10:05 a.m.	Anchoring evidence-based integration of immunization within health services and across sectors (ARRIVE)
10:13 a.m.	Understanding the World Health Organization's global health priorities: document analysis of Global Programmes of Work, 2006-2028
10:21 a.m.	A Novel Conceptual Syndemic Framework for Emergency Department Wait Times: Integrating Systemic and Social Determinants of Health
10:29 a.m.	Prioritizing for Implementation: Lessons from the Smart Choice Process in Advancing Strategi[illegible] Implementation of National Action Plans on Antimicrobial Resistance
10:37 a.m.	Implementing One Health Governance Approaches to Mitigate Antimicrobial Resistance Acros[illegible] Institutional, Social, Economic and Political Contexts
10:45 a.m.	Exploring the Untold Stories of Child Separation through the Context of Homelessness
10:53 a.m.	From Policy to Practice: Evaluating the World Health Organization developed the Package of Essential Noncommunicable Disease Framework for Cardiovascular Disease Management in Ghana
11:01 a.m.	Impact of environmental pollution caused by mining activities on paediatric health in Sudbury, Ontario
10:00 a.m.	ORAL 6: TRANSFORMING SEXUAL AND REPRODUCTIVE HEALTH THROUGH EVIDENCE AND ACTION
10:05 a.m.	MAS por Nosotras: paving the way to improve sexual and reproductive health in female sex workers in Argentina through an Argentinean-Canadian collaboration

10:13 a.m.	Co-creating gender-transformative interventions for adolescent mental, sexual, and reproductive health and rights: Reflections from Niger, Ghana, and Burkina Faso
10:21 a.m.	What do we know about female university students' contraceptive knowledge, attitudes, and practices in Algeria? Insights from an online survey
10:29 a.m.	Scoping Review of Antenatal Care Seeking and Delivery Practices for Pregnant Adolescent Girls in Pakistan
10:37 a.m.	Harnessing the power of youth as change agents for Sexual and Reproductive Health and Rights (SRHR)
10:45 a.m.	Equity Through Action: Scalable Youth-Led Menstrual and Sexual and Reproductive Health Education in Sub-Saharan Africa
10:53 a.m.	Narratives of change show the transformative impact on adolescents of creating and sharing cellphilms about their sexual and reproductive health
11:01 a.m.	Are we on track to realize universal access to sexual reproductive health services and rights: Investigating Gendered Gaps in Contraceptive Use Among sexually active Young People in rural Uganda
11:09 a.m.	Collaboration with Grassroot Religious and Traditional Leaders (RTLs) in Rural Northern Nigeria: Catalysing Positive Attitudinal Change on Social Norms for Uptake of Family Planning Services
11:17 a.m.	Youth Partnership in Adolescents and Youth Sexual Reproductive Health and Rights (AYSRHR) Theory of Change
10:00 a.m.	ORAL 7: PANDEMICS, PREVENTION, AND VACCINE EQUITY: STRENGTHENING GLOBAL HEALTH SECURITY
10:05 a.m.	Laboratory Readiness Assessment for Mpox Response in the ECOWAS Region: Preliminary Findings from a Multi country Survey
10:13 a.m.	Accelerating research during infectious disease outbreaks in Africa: a case study of Ebola vaccine trials
10:21 a.m.	A Unified Regional Response to Emerging Health Threats: How ECOWAS' Coordinated Strategy Mitigated the 2024 Mpox Outbreak in West Africa.
10:29 a.m.	COVID-19 Vaccination Coverage and Public Willingness in Rwanda: A Model for Low-Income Countries
10:37 a.m.	Safety, Efficacy, and Immunogenicity of Mpox Vaccines: A Living Systematic Review and Meta-analysis
10:45 a.m.	Updates on Auditory Outcomes of COVID-19 and Vaccine Side Effects: An Umbrella Review
10:53 a.m.	Facilitators and Barriers to the Functionality of the Vaccine Cold Chain in Rukungiri District, Western Uganda: A Qualitative Inquiry
11:01 a.m.	Canada's pandemic preparedness: moving forward

10:00 a.m.	ORAL 8: SCANNING THE FUTURE: REVIEWS & REFLECTIONS ON AI, INNOVATION, AND GLOBAL HEALTH TRENDS
10:05 a.m.	A Systematic Review of the Role of Artificial Intelligence in Addressing Global Health Threats
10:13 a.m.	Socio-economic Factors Associated with the Adoption of Artificial Intelligence for Healthcare Services in Cameroon: A community-based cross-sectional study
10:21 a.m.	Bridging the Digital Divide in Global Health: A Systematic Review of Strategies for Equitable Telemedicine Access in Low-Resource Regions
10:29 a.m.	Leveraging Machine Learning Algorithms to Assess the Impact of Cervical Cancer Early Detection on Patients Survival
10:37 a.m.	Global Health Innovation: The Impact Of Integrating AI And Digital Health On Health Services Delivery - A Case Study Of World's Largest Virtual Hospital (Seha-Ksa)
10:45 a.m.	Leveraging Machine Learning Algorithms to Assess the Impact of Cervical Cancer Early Detection on Patients Survival
11:30 a.m.	NETWORKING LUNCH
11:30 a.m.	NETWORKING ACTIVITY: Breakout rooms with conversation starters
12:30 p.m.	CCGH 2025 OPENING CEREMONY & WELCOME REMARKS
12:30 p.m.	WELCOME REMARKS & INTRODUCE ELDER EISAN
12:35 p.m.	TERRITORIAL WELCOME & TEACHING
12:47 p.m.	VIDEO: REMARKS FROM GLOBAL AFFAIRS CANADA
12:53 p.m.	KEYNOTE REMARKS
1:00 p.m.	PLENARY: NAVIGATING GOVERNANCE, FINANCING, COLLABORATION & TRUST IN GLOBAL HEALTH
1:00 p.m.	WELCOME & PLENARY INTRODUCTION
1:05 p.m.	KEYNOTE PRESENTATION
1:25 p.m.	PANEL DISCUSSION
1:50 p.m.	OPEN DISCUSSION & AUDIENCE Q&A: MODERATED Q&A SESSION WITH THE AUDIENCE & VIRTUAL ATTENDEES
2:20 p.m.	CLOSING REFLECTIONS
2:30 p.m.	CONCURRENT SYMPOSIA & WORKSHOPS
2:30 p.m.	WORKSHOP 1: Development, evaluation, and analysis of Knowledge, Attitudes and Practice (KAP) questionnaires: From internal consistency to the behavior theory

2:30 p.m.	WORKSHOP 5: Strengthening Health Systems in LMIC Through Responsible AI
2:30 p.m.	SYMPOSIUM 1: Advancing Global Health Preparedness: Collaborative Approaches to Develo Mobile Health Teams in Crisis Settings
2:30 p.m.	SYMPOSIUM - SEP: Rethinking Global Health Education – Leadership, Pedagogy & Possibilities
4:00 p.m.	CONCURRENT ORAL SESSIONS
4:00 p.m.	ORAL 9: POWER, POLITICS & POLICY: WHO REALLY SHAPES GLOBAL HEALTH
4:03 p.m.	Clarifying meanings and significance of solidarity for global health research: results from a qualitative study
4:11 p.m.	Pre-Departure Curriculum and Its Impact on Ethical and Reflexive Experiential Learning and Cultural Humility
4:19 p.m.	The quest for equity in global health is underpinned by neocolonial discourses: an analysis driven by the decolonizing global health movement
4:27 p.m.	Who influences who in setting the health policy agenda? An investigation into the interplay between international organizations and the national government in the Philippines
4:35 p.m.	An International Treaty for a Crispr Bioweapon: Urgency or Overreaction?
4:43 p.m.	Élaboration ou réforme des politiques de santé : compréhension et capacité d'influence des Associations de Professionnels de Santé (APS) au Burkina Faso, en République Démocratiqu du Congo (RDC) et au Sénégal
4:51 p.m.	Semaine nationale de planification familiale au Burkina Faso : Résultats et enseignements tiré après une décennie de mise en œuvre
4:00 p.m.	ORAL 10: COMMUNITY VOICES AND PARTICIPATORY HEALTH RESEARCH IN GLOBAL HEALTH
4:05 p.m.	"He who is not trained by parents is trained by the world": A participatory and gender analysis of parenting in street situations in Kenya to inform the co-creation of a parenting and family violence prevention program
4:13 p.m.	Are women's perceptions integrated in indices used to measure quality of maternal care? A scoping review
4:21 p.m.	Centering African Nova Scotian Voices: Africentric Insights on Gender-Based Violence and Culturally Responsive Service Access
4:29 p.m.	Improving Health Outcomes Through Equitable Urban Environments: A Community-Engaged Model for Equity and Justice in Canada's Greenspaces
4:37 p.m.	"We have to keep fighting so that they can survive": Women-Led Community Mobilization to Mining-Induced Health Deterioration in the Dominican Republic
4:45 p.m.	EmpowHER Kenya: Empowering Youth and Women to Foster Inclusive Leadership in Global Health
4:00 p.m.	ORAL 11: MENTAL HEALTH, DISPLACEMENT, AND THE HUMANITARIAN IMPERATIVE

4:05 p.m.	Burden and Associated Factors of Anxiety and Depression in North Central Nigeria Internally Displaced Persons: A Cross-sectional Study
4:13 p.m.	Advancing Lasting Impact: A Policy Analysis of Long-Term Strategies in Humanitarian Health Aid
4:21 p.m.	Improving Reproductive, Maternal, Neonatal, Child, and Adolescent Health Outcomes through Community Engagement in Conflict-Affected Settings: Insights from Advanced Partnerships in Health project in South Sudan and the Central African Republic
4:29 p.m.	Prévalence et facteurs associés à la détresse psychologique chez les adolescentes et femmes déplacées internes au Burkina Faso
4:37 p.m.	Crise sécuritaire au Burkina Faso : comment assurer la continuité de l'offre des méthodes contraceptives pour les femmes en zones de conflit ?
4:45 p.m.	Trust Rebuilding in Healthcare After Multiple Crises: Lessons from Eastern DRC
4:00 p.m.	ORAL 12: BRIDGING GAPS IN CRISIS: DIGITAL HEALTH SOLUTIONS FOR VULNERABLE POPULATIONS
4:05 p.m.	Connecting Digital Health and Local Realities: Measuring Innovation in Resource-Limited Settings
4:13 p.m.	Evaluating the effectiveness of an AI-based gamified intervention for improving maternal health outcomes among refugees and underserved women in Lebanon
4:21 p.m.	Evaluating the impact of including healthcare providers in an AI-based gamified mHealth intervention for improving maternal health outcomes among disadvantaged pregnant women in Lebanon
6:00 p.m.	CAGH AWARDS CEREMONY & WELCOME RECEPTION

Day 4 - Monday, October 27, 2025

**8:45 a.m.**	**WELCOME REMARKS & HONOURING AFRO-NOVA SCOTIAN HERITAGE ACKNOWLEDGEMENT**
8:45 a.m.	WELCOME REMARKS
8:48 a.m.	**PRESENTATION:** MAKING LEMONADE
**9:00 a.m.**	**PLENARY: HARNESSING INNOVATION: RESULTS FOR WHOM, BY WHOM?**
9:00 a.m.	WELCOME & PLENARY INTRODUCTION
9:05 a.m.	LIGHTNING TALK

9:10 a.m.	LIGHTNING TALK
9:15 a.m.	PANEL DISCUSSION
10:00 a.m.	OPEN DISCUSSION & AUDIENCE Q&A<br>MODERATED Q&A SESSION WITH THE AUDIENCE & VIRTUAL ATTENDEES
10:20 a.m.	CLOSING REFLECTIONS
**10:30 a.m.**	**NETWORKING BREAK**
10:30 a.m.	**NETWORKING ACTIVITY: Virtual Bingo**
**11:00 a.m.**	**CONCURRENT SYMPOSIA & WORKSHOPS**
11:00 a.m.	SYMPOSIUM 3: Strengthening the frontline globally and in Canada: a call for economic and gender justice for community-based health workers
11:00 a.m.	SYMPOSIUM: Adolescent and Indigenous knowledge and beliefs in sexual and reproductive health and mental health research: Experiences from Guatemala, Mexico and Peru
11:00 a.m.	WORKSHOP 3: Creating meaningful community, government and multinational academic partnerships to decolonize Global Health Research
11:00 a.m.	WORKSHOP 4: Leveraging the Power of Civil Society: Building Trust and Resiliency in Sexual and Reproductive Health and Rights
**12:30 p.m.**	**LUNCH & CAGH 2025 ANNUAL GENERAL MEETING**
**2:00 p.m.**	**CONCURRENT SYMPOSIA & WORKSHOPS**
2:00 p.m.	SYMPOSIUM 5 (ROOM 501 & 502): Preparing for the next pandemic: lessons from viral hemorrhagic fevers & the evolution of Safe and Dignified Burials in the Red Cross Red Crescent Movement
2:00 p.m.	SYMPOSIUM 6: Protecting our Collective Future: Renewing Canada's Role in Global Health
2:00 p.m.	SYMPOSIUM 7: Artificial Intelligence and Global Health: Predicting Vulnerability in the Grassroots waste pickers, Ecuador
2:00 p.m.	WORKSHOP 6: World Vision Canada's Urban AHADI SRHR Tanzania Project: Youth Empowerment, Gender Equality and Inclusive Leadership
**3:30 p.m.**	**NETWORKING BREAK**
3:30 p.m.	NETWORKING ACTIVITY: Breakout rooms discussion groups based on interest
**4:00 p.m.**	**CONCURRENT ORAL SESSIONS**

**4:00 p.m.**	**ORAL 13: BEYOND THE BUDGET: GENDER, GENERATIONS & THE POLITICS OF HEALTH FUNDING**
4:05 p.m.	Impact de l'ouverture de nouvelles formations sanitaires et des politiques de gratuité sur l'utilisation des services de santé au Burkina Faso (2013-2023)
4:13 p.m.	Universal health coverage and social health spending efficiency in sub-Saharan Africa
4:21 p.m.	Adolescent Health Financing in West Africa: Evidence on Funding, Availability, and Service Delivery for Sexual, Reproductive, and Mental Health in Burkina Faso, Ghana, and Niger
4:29 p.m.	When it is available, will you pay for it? A Systematic Review and Meta-analysis of Willingness to Pay (WTP) for Malaria Vaccines in Africa
4:37 p.m.	Effect of First Healthcare Contact with healthcare providers on Out-of-Pocket Expenditure in Nigeria: Insights from the National Living Standards Survey 2021
4:45 p.m.	Strengthening the health care system in Ghana through promotion, development and financing of traditional medicine
4:53 p.m.	Why and how did the Centre for Plant Medicine Research ensure access to herbal medicine research and products during the COVID-19 Pandemic?
**4:00 p.m.**	**ORAL 14: GENDER-INCLUSIVE STRATEGIES FOR HEALTH EQUITY AND EMPOWERMENT**
4:05 p.m.	Empowering Isolated Early-Career Midwives: A Gender-Inclusive Mentorship Model to Strengthen Maternal Health Services in Remote Settings
4:13 p.m.	Integrating gender in global tobacco control research
4:21 p.m.	Advocating for equity and resilience through gender-responsive policies for healthcare providers: Findings from the Women in Health and their Economic, Equity and Livelihood statuses during Emergency Preparedness and Response (WHEELER) Study in Kenya.
4:29 p.m.	Évaluation des besoins décisionnels en santé des femmes et des filles dans le Sud Global : Une étude de cas multiples
4:37 p.m.	Starting from the Grassroots: Addressing Gender Norms to Increase Women's Agency in Community Leadership and Decision-Making on SRH in Kaduna, Kano, And Katsina States
4:45 p.m.	Using a gender transformative approach to promote husbands' participation in birth preparedness and complication readiness in The Gambia
4:53 p.m.	An overview of nursing and midwifery leadership, governance structures, and instruments in Africa
**4:00 p.m.**	**ORAL 15: INFECTIOUS DISEASE SURVEILLANCE AND THE POWER OF DATA: NEW EVIDENCE AND ENDURING THREATS**
4:05 p.m.	Tracking changes in the Plasmodium falciparum reservoir in response to sequential malaria control interventions in northern Ghana
4:13 p.m.	What drives the infection: knowledge, attitudes, and practice (KAP) and seroprevalence in a hotspot in Dhaka, Bangladesh

4:21 p.m.	ADJONCTION DES CORTICOSTEROIDES DANS LE TRAITEMENT DE LA TUBERCULOSE CHEZ LES PERSONNES VIVANTES AVEC LE VIH : REVUE SYSTEMATIQUE ET META - ANALYSE
4:29 p.m.	Antibiotic Susceptibility and Resistance Patterns in Bloodstream Infections amongst Hospitalized Patients at J.J. Dossen Hospital, Maryland County - Liberia
4:37 p.m.	Investigating Syphilis-Driven HIV Co-infection Using Blood Donor Records: A 4-Year Retrospective Study at J.J. Dossen Memorial Hospital, Maryland County, Liberia
4:45 p.m.	Prevalence of Hepatitis B Surface Antigen Positivity in Southeast Liberia: Insights from a 3-Year Retrospective Study at James Jenkins Dossen Hospital, Maryland County
4:45 p.m.	"Not just being there...From presence to influence": Rethinking Youth Participation in Global Health Governance Spaces
4:53 p.m.	Prevalence and determinants of post-tuberculosis lung disease in Sub-Saharan Africa: A systematic review and meta-analysis
**4:00 p.m.**	**ORAL 16: MEDTECH FOR IMPACT: FRUGAL AND HIGH-TECH SOLUTIONS TO GLOBAL HEALTH CHALLENGES**
4:05 p.m.	Locally Led MedTech Innovation for Sustainable Global Health: The Frugal Biomedical Innovations Approach
4:13 p.m.	Improving efficiency of the Tuberculosis screening cascade: Utility of an Artificial Intelligence enabled Chest Xray screening in Hotspot communities.
4:21 p.m.	Getting a GRIP on hypertension in Uganda: Isometric handgrip training as a feasible and acceptable intervention for blood pressure control
4:29 p.m.	Acceptability and perceived usability of a non-invasive haemoglobin monitor among community members and health workers in Tanzania
4:37 p.m.	Delivering health for all: Perceptions and impacts of medical delivery drones in remote Madagascar
4:45 p.m.	Newborn Sickle Cell Screening as a PEN-Plus Strategy for Case Finding in Maryland County, Southeast Liberia - A Pilot Study

Day 5 - Tuesday, October 28, 2025

**8:45 a.m.**	**WELCOME REMARKS & HISTORICAL REFLECTIONS FROM HALIFAX**
8:40 a.m.	HOUSEKEEPING REMINDERS
8:45 a.m.	WELCOME REMARKS
8:48 a.m.	CULTURAL PRESENTATION: HALIFAX, MIGRATION, AND PUBLIC HEALTH
8:58 a.m.	THANK STEVEN & INTRODUCE PLENARY SESSION MODERATOR
**9:00 a.m.**	**PLENARY: GLOBAL HEALTH AT A CROSSROADS**
9:00 a.m.	WELCOME, SESSION FRAMING, AND INTRODUCE SPEAKERS

9:07 a.m.	KEYNOTE PRESENTATION - DR. STEVEN HOFFMAN
9:18 a.m.	LIGHTNING QUESTION
9:20 a.m.	KEYNOTE PRESENTATION - DR. AHMED OGWELL OUMA
9:31 a.m.	LIGHTNING QUESTION
9:33 a.m.	KEYNOTE PRESENTATION - PETRA KHOURY
9:43 a.m.	LIGHTNING QUESTION
9:45 a.m.	KEYNOTE PRESENTATION - DR. TOBI KOLLMAN
9:55 a.m.	LIGHTNING QUESTION
9:57 a.m.	OPEN DISCUSSION & AUDIENCE Q&A<br>MODERATED Q&A SESSION WITH THE AUDIENCE & VIRTUAL ATTENDEES
**10:30 a.m.**	**NETWORKING BREAK**
**10:45 a.m.**	**CONCURRENT SYMPOSIA & WORKSHOPS**
10:45 a.m.	SYMPOSIUM 8: Fragile Settings, Unbreakable Resolve: Lifesaving Nutrition Services in Conflict and Chaos
10:45 a.m.	WORKSHOP 7: Future Research Directions in a Changing World
10:45 a.m.	WORKSHOP 8: Leadership féminin et santé : Les CFU, un modèle d'innovation sociale à explorer
**12:15 p.m.**	**CONCURRENT ORAL SESSIONS**
**12:15 p.m.**	**ORAL 17: FROM GLOBAL POLICY TO LOCAL CAPACITY: INNOVATION IN HEALTH SYSTEM SUPPORT AND PATIENT ENGAGEMENT**
12:20 p.m.	Developing a Framework for Evaluation of Global Health Capacity Building in Conflict-Affected Settings
12:28 p.m.	Video-Assisted Education to Facilitate Maternal Engagement in a NICU in Uganda
12:36 p.m.	Gaps in Primary Healthcare Funding and Research in Eastern Africa as Identified by Local Health Professionals
12:44 p.m.	Agents de santé dans les organisations de santé communautaire rurale en Côte d'Ivoire: Perspectives à partir d'une expérience de choix discret
**12:15 p.m.**	**ORAL 18: REDEFINING LEADERSHIP: GENDER, EQUITY AND INCLUSION IN GLOBAL HEALTH**
12:20 p.m.	The future of digital health in Colombia: Experiences of the digital divide of people living with HIV
12:28 p.m.	Experiential Learning Trip Demonstrates the Development of Critical Personal and Professional Competencies

12:36 p.m.	Leveraging Inclusive Leadership in a Changing World: Closing the Women's Health Gap
12:44 p.m.	Centering Community Voices: A Framework Synthesis on the Meaning of Maternal Health for India's Scheduled Tribes and Scheduled Castes
12:52 p.m.	Advancing Gender Equality in Global Health Leadership: A Scoping Review of Barriers and Opportunities for Women in Low- & Middle-Income Countries
1:00 p.m.	Women in White Coats, Are They Safe? A Study on the Caveats of Workplace Safety for Female Healthcare Professionals in Bangladesh
**12:15 p.m.**	**ORAL 19: SEXUAL AND REPRODUCTIVE HEALTH RIGHTS IN THE CONTEXT OF CRISIS AND CHANGE**
12:17 p.m.	From climate change to sexual and reproductive health: Investigating harmful pathways in a shifting funding landscape
12:25 p.m.	Inequalities in effective contraception use in Lebanon: a national telephone study
12:33 p.m.	Transforming HIV Prevention: The Success of an HIV Program in Reducing New Infections Among Pregnant Women in Eswatini
12:41 p.m.	Facteurs associés à l'utilisation du vaccin contre le HPV chez les filles de 9 à 14 ans de la Langue de Barbarie à Saint-Louis au Sénégal en 2024
12:49 p.m.	Migration, Santé reproductive et Résilience des femmes face au changement climatique au Niger, Tillabéri
12:57 p.m.	Laboratory confirmed Puerperal Sepsis and Associated Factors Among Post Delivery Women Admitted at Bugando Medical Centre, Sekou Toure and Sengerema Hospitals in Mwanza ,Tanzania
1:05 p.m.	Exploring the relationship between multi-month dispensing of ART on viral load coverage and suppression among children living with HIV in USAID/PEPFAR-supported country programs in Rwanda, Benin, Côte d'Ivoire, Eswatini, Kenya, Malawi, Senegal, Togo, Zimbabwe, Burkina Faso, the Democratic Republic of the Congo, Ghana, Lesotho, Mali, South Sudan, Uganda, Burundi
**12:15 p.m.**	**ORAL 20: AI, BIG DATA & PREDICTIVE HEALTH IN GLOBAL CONTEXTS**
12:20 p.m.	Technology Enables Proactive Care Transition: The Experience and Impact of the IMPALA Patient Monitoring System in a Rwandan Neonatal Intensive Care Unit
12:28 p.m.	HarThali: Harnessing Blockchain and AI to Transform Food Insecurity and Improve Malnutrition in Dharavi, India
12:36 p.m.	Classifying land use using LiDAR data and satellite imagery to assess the association between green space and diabetes
12:44 p.m.	Leveraging Mapping Technology to Identify Service Gaps: Addressing Food Insecurity and Food Bank Access
**1:15 p.m.**	**CLOSING LUNCH & CCGH 2025 AWARDS**
1:15 p.m.	WELCOME & CLOSING REFLECTIONS
1:20 p.m.	PRESENT CCGH 2025 AWARDS (4)
1:30 p.m.	INTRODUCE CCGH 2026 CO-CHAIRS

1:35 p.m.	ANNOUNCE 2026 LOCATION
1:40 p.m.	CLOSING REMARKS & SURVEY REMINDER
**2:15 p.m.**	**CONCURRENT ORAL SESSIONS**
**2:15 p.m.**	**ORAL 21: BUILDING TRUST AND INCLUSION IN GLOBAL AND LOCAL HEALTH GOVERNANCE**
2:17 p.m.	Caught between Hope and Frustration: The Complex Realities of NCD Caregivers amid Collaboration and (Mis)trust" in an LMIC Context
2:25 p.m.	Feminist theories in global health governance research: a literature review
2:33 p.m.	Advancing HIV Response in Rwanda: Funding models, achievements, and future directions
2:41 p.m.	From Margin to Mainstream: Ethnography of Local Health Systems in Nepal
2:49 p.m.	Patient Agency in Integrating Alternative Medicine with Orthodox Care - Insights from Ghana, Niger and Burkina Faso
2:57 p.m.	Ampleur et Tendances de la Fuite des Cerveaux dans le secteur Médical (FCM) en provenance de l'Afrique : nouvelles évidences basées sur la migration des médecins et des infirmiers
**2:15 p.m.**	**ORAL 22: YOUTH EMPOWERMENT STRATEGIES FOR HEALTH, EQUITY AND CHANGE**
2:17 p.m.	Youth empowerment amid compounding crises: An evaluative study of a youth-centred community development program in El Salvador
2:25 p.m.	Empowering marginalized adolescent girls and young women to advocate for their health and well being
2:33 p.m.	Disparities in India's Fertility Transition: The Role of Youth Empowerment in Achieving Replacement-Level Fertility
2:41 p.m.	Empowering Adolescents in Rural Communities: A Peer-Led Approach to Addressing Social and Health Challenges
2:49 p.m.	Les structures des jeunes : un levier pour promouvoir la santé sexuelle et reproductive des adolescents au Niger
2:57 p.m.	Engagement fort de l'Association Togolaise pour le Bien Être Familial (ATBEF) pour une jeunesse sans grossesse précoces en milieu scolaire
3:05 p.m.	Empowering Marginalised Youth through the Intersectional Community Scorecard: Advancing Gender Equity and Social Inclusion in SRHR
**2:15 p.m.**	**ORAL 23: OUTBREAKS AND OPERATIONAL READINESS: RESPONDING TO EPIDEMICS AND STRENGTHENING SURVEILLANCE**
2:20 p.m.	Exploring the Role of Experiential Learning in Tanzania on the Understanding of One Health
2:28 p.m.	Marburg Virus Disease in Rwanda during the first 10 days: A Critical Call for Global Action to Rethink, Reposition, and Respond
2:36 p.m.	Measles outbreak investigation in Moroto District, North Eastern Uganda, March to July 2024

**2:15 p.m.**	**ORAL 24: INCLUSIVE DIGITAL CARE: ADHERENCE, AND EQUITY IN MATERNAL, ADOLESCENT, SRH AND COMMUNITY HEALTH**
2:20 p.m.	Evaluating the effectiveness of Essential Coaching for Every Mother in Tanzania (ECEM-TZ): A randomized controlled trial
2:28 p.m.	Health behaviours and risk factors associated with cardiovascular disease in a multi-national South Asian population
2:36 p.m.	Infoadojeunes : une innovation numérique au service de l'offre de services conviviaux et de l'éducation sexuelle complète adaptée aux jeunes
2:44 p.m.	Harnessing Innovation for Health Equity: Scaling Frugal Innovations in Homa Bay County's Underserved Communities
**3:15 p.m.**	**CONCURRENT ORAL SESSIONS**
**3:15 p.m.**	**ORAL 25: ADVANCING EQUITY THROUGH HEALTH GOVERNANCE: GLOBAL PERSPECTIVES, LOCAL IMPACT**
3:20 p.m.	More Than a Label: Reframing 'Gluten-Free' as a Global Health Commitment
3:28 p.m.	Rotating Savings and Credit Associations (ROSCAs) and Health Equity in Urban and Rural South Africa: A Gendered Analysis of Food Security and Obesity Using Community-Based Financial Networks
3:36 p.m.	Évaluation stratégique du Plan Stratégique National de Santé Communautaire du Sénégal (PSNSC 2020-2024) : une approche multi sources et territorialisée
3:44 p.m.	Implications of District and Parish Level Decentralization of Health Programs in Uganda: A Case Study of the National Malaria Control Program
**3:15 p.m.**	**ORAL 26: RETHINKING YOUTH PARTICIPATION, INFLUENCE, AND LEADERSHIP**
3:17 p.m.	Youth community engagement in knowledge mobilization activities
3:25 p.m.	Centering Youth Voice in Adolescent Health Systems: A Gender-Sensitive Visual Analysis from Ghana
3:33 p.m.	Scoping Study on the Health of Unsheltered Youth in Latin America and the Caribbean
3:41 p.m.	Raising and Increasing Voice, Participation, and Leadership of Adolescents in Adolescent Mental, Sexual, and Reproductive Health Design, Programming, and Implementation Using Theatre for Development in West Africa
3:49 p.m.	Youth-Led Advocacy in Action: Championing Comprehensive Sexuality Education as a Stand-Alone Subject in Sierra Leon
3:57 p.m.	Civil Society for Implementation Planning: Advancing Health Policy in Uganda
4:05 p.m.	Access to medical and public health studies funding to boost youth empowerment as change agents in global health

**3:15 p.m.**	**ORAL 27: FRONTLINE REALITIES: WOMEN’S HEALTH, WORKFORCE SAFETY, AND EQUITY IN CHALLENGING CONTEXTS**
3:20 p.m.	Améliorer la surveillance épidémiologique pour une détection précoce en RDC
3:28 p.m.	Occupational Health and Safety Training for Immigrant Workers: A Scoping Review of Access, Use, and Retention Factors
3:36 p.m.	Knowledge, attitudes, and practices related to respiratory infections among working women living in urban informal settlements in Dhaka and Gazipur, Bangladesh
3:44 p.m.	Outcomes of nurses led community-based intensive-phase multi-drugs resistant tuberculosis (MDR-TB) treatment pilot in Montserrado, County, Liberia
**3:15 p.m.**	**ORAL 28: EDUCATING FOR CHANGE: DIGITAL TOOLS IN HEALTH LITERACY, SRHR, AND CAPACITY BUILDING**
3:20 p.m.	LEAPing into Digital Health: Harnessing mHealth to Strengthen Community Health Worker Capacity for RMNCAH Education and Services
3:28 p.m.	Transformative Pedagogy in Interprofessional Health Education: Catalyzing Anti-Racist Practice and Cultural Responsiveness
3:36 p.m.	E-learning atbef.org : une innovation de formation d'Education Sexuelle Complète en ligne pour la mise à l'échelle de l'ESC au Togo
